# Selenium Donor Inhibited Hepatitis B Virus Associated Hepatotoxicity via the Apoptosis and Ferroptosis Pathways

**DOI:** 10.1155/2023/6681065

**Published:** 2023-08-30

**Authors:** Jingdong Shi, Zhen Liu, Weina Li, Di Wang

**Affiliations:** ^1^General Surgery Department, Beijing Tian Tan Hospital, Capital Medical University, Beijing 100050, China; ^2^The Eighth Medical Center, Chinese PLA General Hospital, Beijing 100091, China; ^3^Medical Research and Laboratory Diagnostic Center, Jinan Central Hospital, Cheeloo College of Medicine, Shandong University, Jinan 250013, Shandong, China

## Abstract

**Methods:**

The serum selenium level was determined in 45 patients with HBV-positive HCC (HBV^+^-HCC group), 45 patients with chronic hepatitis B virus infection (CHB group), and 45 healthy cases (HC group). The sodium selenite (Na_2_SeO_3_)-treated HepG2.2.15 cells were used to observe the regulatory role of selenium on HBV replication. D-GalN/erastin-added HL7702 was used to determine the regulatory roles of Na_2_SeO_3_ on hepatotoxicity or hepatocyte ferroptosis. The wild-type (WT) C57BL/6 mice and HBx-Tg mice were received lipopolysaccharide (LPS)/D-GalN, together with or without Na_2_SeO_3_ administration for indicated period. Following euthanasia, the blood and liver tissue samples were collected, and specific markers were evaluated subsequently.

**Results:**

The serum selenium level was downregulated in patients with HBV-positive HCC (HBV^+^-HCC group) (57.2 ± 22.5 *μ*g/L vs. 91.8 ± 43.9 *μ*g/L, *P* < 0.001), and its higher level could provide a better prognosis in these patients. The treatment using Na_2_SeO_3_, a selenium donor, at high concentration (5 *μ*M), suppressed the HBV replication by about 50% in HepG2.2.15 cells (*P* < 0.001), through promoting apoptotic cell death and inhibiting cellular inhibitor of apoptosis proteins (cIAPs). In addition, low-dose (500 nM) Na_2_SeO_3_ could almost totally reversed the hepatotoxicity induced by hepatitis B virus X protein (HBx) (*P* < 0.001), which were the main causes of HCC in patients. Studies at the cellular levels showed that low-dose Na_2_SeO_3_ inhibited the HBx-related hepatotoxicity by blocking ferroptosis, and glutathione peroxidase 4 (GPX4) mediated this regulatory role. Mice model results confirmed that the treatment with Na_2_SeO_3_ could mitigated LPS/D-GalN-induced hepatic injury through ferroptosis pathways.

**Conclusion:**

Selenium regulated the dual cell death in different HCC stages via different signaling pathways, which could partly explain the anti-HBV and anti-HCC properties of selenium.

## 1. Introduction

Chronic infection caused by the hepatitis B virus (HBV) is a major risk factor for hepatocellular carcinogenesis (HCC). About 350 million people globally are chronically infected with HBV, and chronic HBV infection accounts for at least 50% cases of HCC worldwide [1–3]. Mechanistically, specific proteins encoded by HBV, including hepatitis B virus X protein (HBx), and members express proteins, make cell transcription and proliferation disorder, and make hepatocytes sensitive to carcinogens [4]. Although the universal vaccination of newborns has led to a decline in HCC, many challenges, such as difficulties in accessing treatment and the emergence of drug-resistant strains, still exist in dealing with HBV and associated cancers [5].

Selenium is an essential element found in mammals, and selenium deficiency may increase the risk of a variety of diseases including cancer [6]. Studies revealed that selenium was beneficial in terms of antiviral therapy against various virus types such as coronavirus disease-19 [7], human immunodeficiency virus, hepatitis C virus (HCV), and HBV [6]. Although the mechanism is still not clear, we can infer that selenium might restrict HBV replication and its related hepatocarcinogenesis, since selenium could regulate host immune response and had the antioxidant effects, which lead to decreased viral replication and cellular damage.

The mechanism of host immune response on HBV-associated HCC is complex. In the early stage of infection, infected hepatocytes release antigens, activating antigen-presenting cells to initiate an antiviral response. Cell death processes are then triggered to eliminate infected cells and release tumor-associated antigens. As the infection intensifies, excessive cell death would cause liver injury and hepatotoxicity, which are recognized as the key carcinogenic mechanisms of HBV [8]. When the tumor occurs, HBV in turn inhibits tumor cell death, thereby promoting cell proliferation and metastasis [9]. Therefore, cell death is closely related to the occurrence and development of HBV-associated HCC, and recent reports have also confirmed that targeting the cell death pathway is an effective strategy for inhibiting HBV-associated HCC [9].

Many types of programmed and nonprogrammed cell deaths have been reported, including apoptosis, autophagic death, necrosis, pyroptosis, and ferroptosis [10, 11]. The signaling pathways of these cell death types are different and usually crosstalk [10, 11]. The literatures have reported that selenium can regulate various cell death types, so we speculate that the specific cell death pathway may mediate the regulation of selenium on HBV-associated HCC [6].

HepG2.2.15 cell line is a kind of HBV infected HCC cells, which can simulate HBV replication in the host. HL7702 cells with D-GalN is a recognized liver cell injury model. HBx-Tg mice are transgenic mice that express HBx protein, which are widely used to study the function of HBV-associated HCC development. Therefore, we tried to explore the effect of selenium on HBV infection and its related hepatotoxicity in terms of regulatory cell deaths based on these cell model and mice model.

## 2. Materials and Methods

### 2.1. Clinical Participants

All enrolled chronic hepatitis B virus infection (CHB group) patients were characterized as HBsAg-positive >6 months, no antibody to hepatitis B surface antigen (anti-HBs) existed, persistent or intermittent elevation of ALT and (or) AST levels, and with no evidence of cirrhosis or carcinoma by imaging and laboratory testing. HCC patients with anti-HBs existed were defined as HBV-related HCC (HBV^+^-HCC group). In this study, 45 patients in HBV^+^-HCC group, 45 patients in CHB group, and 45 healthy cases (HC group) were enrolled. We excluded the patients with the following factors: (A) HBV DNA negative patients, indicating that the virus is in inactive period; (B) patients with hepatitis A, hepatitis C, hepatitis D, hepatitis E, Epstein–Barr virus, cytomegalovirus, or human immunodeficiency virus; (C) patients with advanced liver disease such as liver cirrhosis or acute-on-chronic liver failure; (D) previous antiviral or other intervention treatments; (E) age <18 or gravida; and (F) refusal to participate or drop out during follow-up.

The blood samples were collected before surgery from all the HBV^+^-HCC group patients to monitor certain markers. The blood samples from patients belonging to the CHB and HC groups were collected at the same time. The serum selenium level was determined using a graphite atomic absorption spectrophotometer [12, 13]. The liver function indexes including aspartate aminotransferase (AST), alanine transaminase (ALT), total bilirubin (TBIL), and albumin (ALB) were recorded to assess the hepatotoxicity.

Indications of antiviral therapy are generally based mainly on the combination of serum HBV DNA levels, serum ALT levels, and severity of liver diseases according to previous reported guideline [14]. Antiviral therapy included lamivudine, entecavir, or telbivudine monotherapy.

The clinical study was approved by the ethics committee of the Tiantan hospital (approval number: 2022-146) and informed consent was obtained from all patients before participation. All the experiments were carried out according to principles of Helsinki Declaration.

### 2.2. Cell Culture

HepG2.2.15, HepG2, and HL7702 cells were cultured in Dulbecco's Modified Eagle Medium (Invitrogen). The cultured medium was supplemented with 10% fetal bovine serum, 100 IU/mL penicillin, and 100 *µ*g/mL streptomycin. All cells were incubated in a humidified 5% CO_2_ atmosphere at 37°C before use.

### 2.3. HBV Load Determination

The levels of HBV surface antigen (HBsAg), e antigen (HBeAg), HBV mRNA, and HBV DNA, which could reflect the HBV replication and transcription, were determined by the methods suggested in previous studies [15]. Generally, HBsAg and HBeAg levels in the supernatant were determined using the enzyme-linked immunosorbent assay (ELISA) HBV test kits (KHB) following the manufacturer's protocols. HBV mRNA or HBV DNA levels were determined by quantitative polymerase chain reaction or quantitative reverse transcription-polymerase chain reaction.

### 2.4. HBV Reporter Gene Plasmid Construction

The HBV reporter gene plasmids were constructed by the methods suggested in a previous study [15]. The *Xba*l-*Hind*III fragments containing the surface promoter I (SPI), surface promoter II (SPII), and core promoter (CP) were inserted into the *Nhe*I-*Hind*III site of the pGL3-Basic vector (Promega, WI, USA). They were labeled SPI-Luc, SPII-Luc, CP-Luc, and XP-Luc, respectively.

### 2.5. Dual-Luciferase Assay

HepG2 cells were transiently transfected with reporter vectors besides certain treatments. The cells were collected for luciferase assay, which was performed using the dual-luciferase reporter assay system, 48 hr after transfection (Promega) following the manufacturer's protocol.

### 2.6. MTT Assay

3-(4,5-Dimethylthiazole-2-yl)-2,5-diphenyltetrazolium bromide (MTT) was used to determine the cell viability. Briefly, the cells cultured in 96-well plates were treated with MTT solution for 3 hr, followed by dimethyl sulfoxide, and then placed at room temperature for 15 min. Finally, the ELISA microplate reader (Dynex, USA) was used to measure the absorbance at 570 nm (optical density (OD) value). The cell number was calculated using the corresponding OD values on the standard curves.

### 2.7. Western Blot Analysis

The protein extracts were prepared in a radioimmunoprecipitation assay lysis buffer (Pierce; Thermo Fisher Scientific). The protein estimation was performed using bicinchoninic acid protein assay kit (Pierce, Thermo Fisher Scientific). Proteins were separated using SDS-PAGE and transferred onto PVDF membrane (Pierce, Thermo Fisher Scientific). After blocking, the blots were incubated with primary antibodies at 4°C overnight, followed by the incubation using secondary antibodies. The protein bands were visualized using enhanced chemiluminescence and film exposure.

### 2.8. Flow Cytometric Apoptosis Analysis

The cells of certain groups were harvested using trypsin, resuspended in phosphate buffered saline (1 × 10^6^ cells/mL), and analyzed for cell apoptosis rate via flow cytometry using Annexin V-FITC/PI method.

### 2.9. Ferroptosis Marker Assay

Glutathione peroxidase (GPx) activities were determined using H_2_O_2_ as the substrate based on a previous study [16]. The reaction was monitored indirectly as the oxidation rate of nicotinamide adenine dinucleotide phosphate at 240 nm for 3 min. The quantification of the oxidative stress marker malondialdehyde (MDA) was performed using a lipid peroxidation (MDA) assay kit (Abcam, Cambridge, UK). The relative iron level in cell or tissue extracts was observed using an iron assay kit (ab83366; Abcam). The relative GPX4 levels in serum were determined using the ELISA kits (FT-P36760R; Fantaibio, Shanghai, China). All the aforementioned kit-based experiments were conducted following the manufacturer's protocols.

### 2.10. Animal Study

The wild-type (WT) C57BL/6 mice (SM-001; Shanghai, China) and HBx-Tg mice (NM-TG-00003; Shanghai, China) were purchased from Shanghai Model Organisms Center, Inc. (https://www.modelorg.com/en/). Animals were housed and bred according to the Care and Use of Laboratory Animals guidelines of the US National Institute of Health, under controlled temperature (23°C ± 2°C), light–dark cycle (12 hr light/12 hr dark) and relative humidity (50% ± 10%). Food and water were provided ad libitum. The animal study was approved by the ethics committee of the Tiantan hospital (approval number: 2022-146).

This study did not use female mice to avoid interference with experimental results caused by changes in hormone levels during the physiological cycle of female mice. The 6–8-week-old male WT or HBx-Tg mice, about 20 g weight were randomly divided into control, lipopolysaccharide (LPS)/D-GalN, and LPS/D-GalN^+^ sodium selenite (Na_2_SeO_3_) or Fer-1 groups, based on the random sequence generated by Excel (version 2013). Each treatment group was composed of eight mice, which were kept in a cage. This sample size was determined on the basis of empirical data from pilot or previous experiments, which were sufficient to detect differences as small as 10% using the statistical methods described. Totally, 64 mice were used for this experiment. The LPS/D-GalN group was intraperitoneally administered with LPS (0.25 mg/kg) and D-GaIN (400 mg/kg). The WT or HBx-Tg mice were given Na_2_SeO_3_ (3 mg/kg) 1 hr prior to D-GalN and LPS administration. Then, the mice received 10 mg/kg Fer-1 1 hr prior to D-GalN and LPS injection. After 5 hr of LPS/D-GalN injection, the mice were sacrificed by CO_2_ inhalation, so that to reduce pain, suffering, and distress. The mice were placed into carbon dioxide (CO_2_) box and 100% CO_2_ was imported. The following ratios of CO_2_/O_2_% vol/min were applied for induction 5/95% vol/min and for euthanasia 100/0% vol/min. The blood was collected from the abdominal aorta, and the liver was dissected. The protein and total RNA were extracted immediately from the liver, numbered, and frozen at −80°C. The specific indicators were subsequently tested by another experimenter in a blinded fashion. Liver injury indicators, including ALT, AST values, and the injury grade reflected by H&E staining, are considered as the primary experimental outcomes; MDA and iron levels are considered as secondary experimental outcomes.

### 2.11. Statistical Analysis

The statistical analysis was conducted using the SPSS Statistics 19.0 software (SPSS, IL, USA). All data were shown as the means ± standard deviation. The statistical analyses were performed using the Student *t*test. The *P* value <0.05 indicated a statistically significant difference.

## 3. Results

### 3.1. Serum Selenium Levels in Patients with HBV-Related HCC

**Figure 1:**
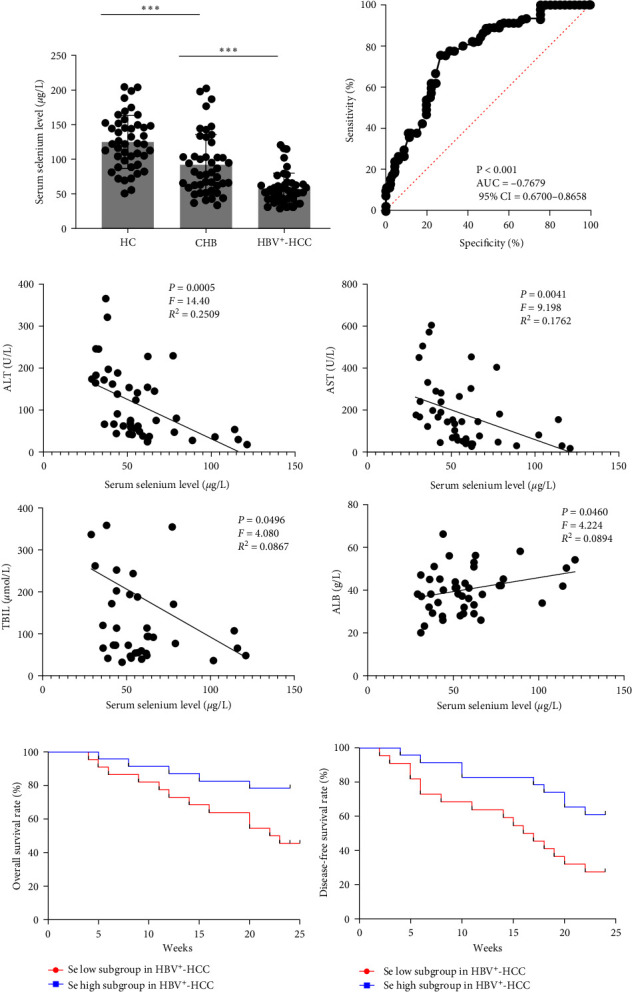
Serum selenium levels in HBV^+^-HCC patients, CHBs patients and the healthy controls. (a) Comparing the serum selenium level among HBV^+^-HCC patients, CHBs patients, and the healthy controls. (b) The ROC curve for serum selenium levels in relation to the HBV^+^-HCCs and CHBs. (c) Linear regression analysis between serum selenium levels and serum ALT, AST, TBIL, and ALB levels. (d) Overall survival curve and (e) disease-free survival curve of HBV^+^-HCC patients with different serum selenium level. HBV^+^-HCC: HBV positive HCC patients; CHBs: chronic hepatitis B virus infection patients; HCs: healthy controls; Se: selenium;  ^*∗*^*P* < 0.05;  ^*∗∗*^*P* < 0.01;  ^*∗∗∗*^*P* < 0.001.

The detailed demographics of all the patients are listed in Table 1. We tested the serum selenium levels in all the patients (patients in the HBV^+^-HCC group were examined before surgery). We found that the HBV^+^-HCC group showed lower serum selenium levels than the CHB group (57.2 ± 22.5 *μ*g/L vs. 91.8 ± 43.9 *μ*g/L, *P* < 0.001), and patients in both HBV^+^-HCC and CHB groups had lower serum selenium levels than those of HCs (Figure 1(a)). Using the receiver operating characteristic curve (Figure 1(b)), we found that the serum selenium levels could distinguish between HBV^+^-HCC and CHB groups, with the area under the curve value as 0.7679 (95% confidence interval = 0.6700–0.8658).

Subsequently, patients in the HBV^+^-HCC group were further investigated for the relationship between their clinical characteristics and serum selenium levels. The hepatotoxicity index, such as AST, ALT, and TBIL levels, was negatively correlated with the serum selenium levels, and ALB levels had significant positive correlations with serum selenium levels (Figure 1(c)). Using the median selenium value as the cutoff, we divided the HBV^+^-HCC group patients into serum selenium low and high groups. A significant negative correlation between the serum selenium levels with tumor size and lymphatic metastasis was found in patients in the HBV^+^-HCC group (the correlation with tumor stage was not statistically significant) (Table 2). Furthermore, the ratio of patients requiring antiviral treatment was higher in serum selenium low group, in both CHB and HBV^+^-HCC cases (Table 2). We followed up with the HBV^+^-HCCs group patients for 2 years. The Kaplan–Meier curve and the log-rank test showed that HBV^+^-HCC group patients with low serum selenium phenotype had an unfavorable overall survival prognosis (Figure 1(d)) and worse disease-free survival (Figure 1(e)).

### 3.2. Selenium Donor Suppressed HBV Replication in HepG2.2.15 Cells via Apoptosis

**Figure 2:**
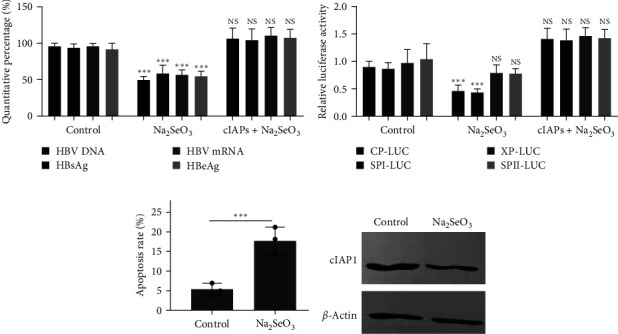
Effects of Na_2_SeO_3_ on HBV replication in HepG2.2.15 cells. (a) HepG2.2.15 cells were transiently transfected with plasmid pcDNA3.1-cIAPs or pcDNA3.1-NC as empty control for 24 hr, and then were treated with Na_2_SeO_3_ at 5 *μ*M for another 24 hr, and then the cells were harvested and HBV DNA, HBV mRNA HBsAg, and HBeAg quantities were determined. (b) HepG2 cells were transiently transfected with reporter vectors of HBV promoters CP-Luc, XP-Luc, SPI-Luc, and SPII-Luc, along with plasmid pcDNA3.1-cIAPs or pcDNA3.1-NC for 24 hr, and then were treated with Na_2_SeO_3_ at 5 *μ*M for another 24 hr, and then the luciferase assay was performed. (c) The histogram presents the statistical values of apoptosis rate. (d) HepG2.2.15 cells were treated with Na_2_SeO_3_ at 5 *μ*M for 24 hr, and then the cells were harvested and the cIAPs levels were determined by western blotting assay.  ^*∗*^*P* < 0.05;  ^*∗∗*^*P* < 0.01;  ^*∗∗∗*^*P* < 0.001.

We used HepG2.2.15 cells, which were formed by transfecting the receptor cell HepG2 with two recombinant plasmids of the whole HBV DNA gene connected head to tail [16], to determine the effect of selenium on HBV replication *in vitro*. We found that a selenium donor, Na_2_SeO_3_, decreased the HBV DNA, HBV mRNA, HBsAg, and HBeAg levels by about 50% at a concentration of 5 *μ*M in HepG2.2.15 cells (Figure 2(a), *P* < 0.001). This finding was similar to the one reported by Cheng et al. [17]. We next transfected HepG2 cells with specific HBV promoter reporters to determine the effects of Na_2_SeO_3_ on HBV. As shown in Figure 2(b), Na_2_SeO_3_ significantly inhibited the CP-Luc and XP-Luc activities, but had little effect on SPI-Luc and SPII-Luc (Figure 2(b)).

Previous studies demonstrated that Na_2_SeO_3_ mainly triggered apoptotic cell death [18], but apoptosis enhancement was found to facilitate HBV elimination [19]. A previous study demonstrated that the cellular inhibitor of apoptosis proteins (cIAPs) impaired the clearance of HBV, and targeting cIAPs might be a new strategy for treating chronic HBV infection [19]. In this study, we found that the HepG2.2.15 cell apoptosis rate increased after treatment with 5 *μ*M Na_2_SeO_3_ (Figure 2(c)), and the cIAP1 levels were downregulated (Figure 2(d)). The overexpression of cIAP1 could reverse the HBV inhibition by Na_2_SeO_3_ (Figures 2(a) and 2(b)). The aforementioned results indicated that selenium donor suppressed HBV replication in HepG2.2.15 cells via the apoptosis pathway.

### 3.3. Selenium Donor Inhibited the HBx-Related Hepatotoxicity in Normal Hepatocytes Independent of Apoptotic Cell Death

**Figure 3:**
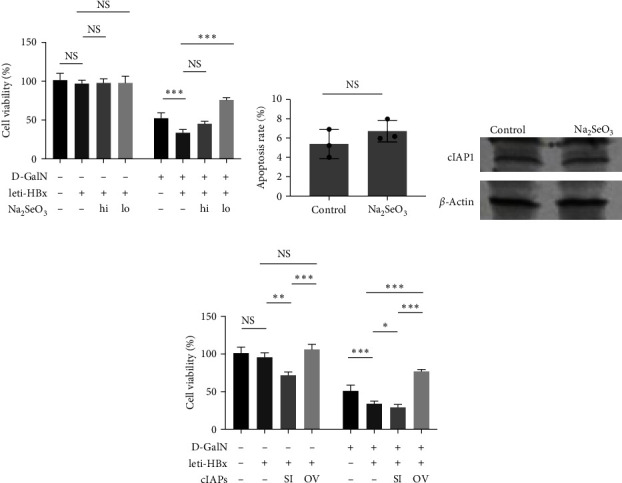
Effects of Na_2_SeO_3_ on HBx-related hepatotoxicity. (a) HL7702 cells were transduced with leti-HBx or leti-NC as empty control for 24 hr, and then were treated with 50 mM D-GalN, along with Na_2_SeO_3_ at certain dose (hi: 5 *μ*M; lo: 500 nM) for another 6 hr, and then the cell viability was monitored by MTT assay. (b) The histogram presents the statistical values of apoptosis rate. (c) HL7702 cells were treated with 50 mM D-GalN, along with Na_2_SeO_3_ at 500 nM for 6 hr, and then the cells were harvested and the cIAPs levels were determined by western blotting assay. (d) HL7702 cells were transduced with leti-HBx or leti-NC as empty control for 24 hr, and then were transiently transfected with plasmid pcDNA3.1-cIAPs or cIAPs siRNA for 24 hr, and then the cell viability was monitored by MTT assay.  ^*∗*^*P* < 0.05;  ^*∗∗*^*P* < 0.01;  ^*∗∗∗*^*P* < 0.001.

The clinical data also indicated that the high selenium level was associated with less HBV-induced hepatotoxicity (the AST and ALT levels as indicators). HBx-related hepatotoxicity is the key inducer of tumors in patients with early-stage HBV-positive HCC [20, 21]. This study constructed a hepatotoxicity cell model using HL7702 cells by adding D-GalN based on the methods suggested in previous studies [21]. This cell model was additionally treated with or without lev-HBx (HBx overexpression lentivirus), along with or without Na_2_SeO_3_. HBx enhanced the hepatotoxicity of D-GalN against HL7702 cells per the previous findings [8]. Na_2_SeO_3_ at a concentration of 5 *μ*M had no effect on cell viability in this model, but low-dose Na_2_SeO_3_ (500 nM) almost totally reversed the hepatotoxicity enhancement induced by HBx (Figure 3(a)). At this dose, Na_2_SeO_3_ could not impact the cell apoptosis rate and cIAPs levels in HL7702 cells (Figure 3(b) and 3(c)). Moreover, cIAP small interfering RNA, which was used to induce apoptosis, increased the cytotoxicity level induced by HBx, whereas the cIAP overexpression decreased the cytotoxicity (Figure 3(d)). These data indicated that selenium donor inhibited the HBx-related hepatotoxicity in normal hepatocytes independent of apoptotic cell death.

### 3.4. Selenium Donor Inhibited the HBx-Related Ferroptosis

**Figure 4:**
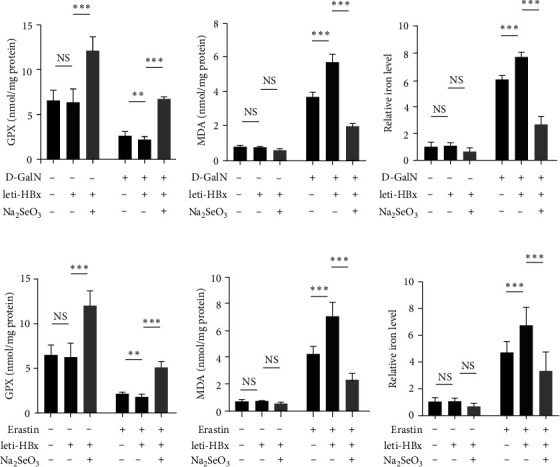
Effects of Na_2_SeO_3_ on HBx-related ferroptosis. (a–c) HL7702 cells were transduced with leti-HBx or leti-NC as empty control for 24 hr, and then were treated with 50 mM D-GalN, along with Na_2_SeO_3_ at 500 nM for 6 hr, and then the cells were harvested and the (a) GPx activities; (b) MDA levels; and (c) relative iron levels were determined. (d, e) HL7702 cells were transduced with leti-HBx or leti-NC as empty control for 24 hr, and then were treated with Erastin at 10 *μ*M, along with Na_2_SeO_3_ at 500 nM for 6 hr, and then the cells were harvested and the (d) GPx activities; (e) MDA levels; and (f) relative iron levels were determined.  ^*∗*^*P* < 0.05;  ^*∗∗*^*P* < 0.01;  ^*∗∗∗*^*P* < 0.001.

A selenoprotein family, comprising GPxs, was found to be activated by selenium [16]. In this study, we found that HBx treatment decreased the GPx activity, and Na_2_SeO_3_ treatment at a concentration of 500 nM significantly induced the GPx activities in D-GalN-treated HL7702 cells (Figure 4(a)). Moreover, the MDA and iron levels were increased by HBx and decreased after treatment with 500 nM Na_2_SeO_3_ (Figures 4(b) and 4(c)). The GPx activity (negative correlation marker) and MDA and iron levels (positive correlation markers) were the markers of the ferroptosis process [10, 11]. Therefore, we hypothesized that HBx might promote ferroptosis, while selenium might inhibit ferroptosis-related cell death. To prove our hypothesis, we used a ferroptosis activator, erastin, to induce HL7702 cell ferroptosis and found similar regulatory effects of HBx and Na_2_SeO_3_ on these ferroptosis markers (the GPx activity, and MDA and iron levels) (Figure 4(d)–4(f)). These findings indicated that selenium donors could inhibit HBx-related ferroptosis.

### 3.5. Na_2_SeO_3_–GPX4 Axis Inhibited the HBx-Related Hepatotoxicity via Ferroptosis

**Figure 5:**
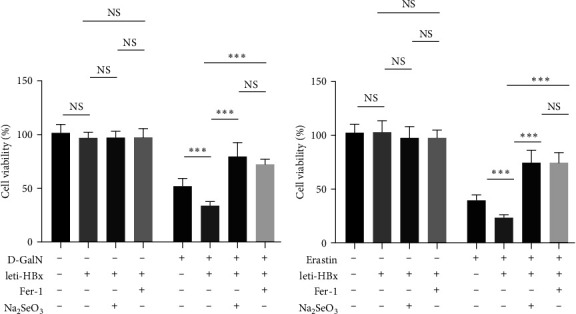
Similar function of Fer-1 and Na_2_SeO_3_ on the HBx-related hepatotoxicity. (a) HL7702 cells were transduced with leti-HBx or leti-NC as empty control for 24 hr, and then were treated with 50 mM D-GalN, along with Na_2_SeO_3_ at 500 nM or Fer-1 at 1 *μ*M for 6 hr, and then the cell viability was monitored by MTT assay. (b) HL7702 cells were transduced with leti-HBx or leti-NC as empty control for 24 hr, and then were treated with Erastin at 10 *μ*M, along with Na_2_SeO_3_ at 500 nM or Fer-1 at 1 *μ*M for 6 hr, and then the cell viability was monitored by MTT assay.  ^*∗*^*P* < 0.05;  ^*∗∗*^*P* < 0.01;  ^*∗∗∗*^*P* < 0.001.

Liu et al. [8] reported that HBx facilitated ferroptosis in hepatocytes, which might play a significant role in hepatotoxicity. We added a ferroptosis blocker, Fer-1, in the D-GalN-treated HL7702 cells or in the erastin-treated HL7702 cells. The results indicated that the effect of Fer-1 on hepatotoxicity was similar to that of 500 nM Na_2_SeO_3_ treatment (Figures 5(a) and 5(b)). This result confirmed that a decrease in ferroptosis could inhibit HBx-related hepatotoxicity.

**Figure 6:**
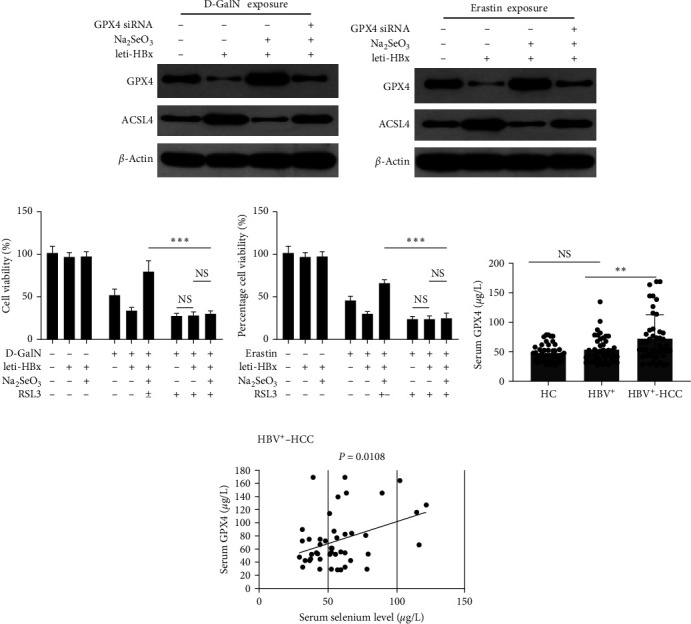
Na_2_SeO_3_-GPX4 axis inhibits the HBx-related hepatotoxicity through ferroptosis dependent manner. (a and b) HL7702 cells were transduced with leti-HBx or leti-NC as empty control for 24 hr, and then were treated with (a) 50 mM D-GalN or (b) Erastin at 10 *μ*M, along with Na_2_SeO_3_ at 500 nM for 6 hr, and then the cells were harvested and the GPX4 and ACSL4 levels were determined by western blotting assay. (c and d) HL7702 cells were transduced with leti-HBx or leti-NC as empty control for 24 hr, and then were treated with (c) 50 mM D-GalN or (d) Erastin at 10 *μ*M, along with Na_2_SeO_3_ at 500 nM, with or without RSL3 at 3 *μ*M for 6 hr, and then the cell viability was monitored by MTT assay. (e) Comparing the serum GPX4 levels among HBV^+^-HCC patients, CHBs patients and the healthy controls. (f) Correlation between the serum selenium levels and serum GPX4 levels in HBV^+^-HCC patients.  ^*∗*^*P* < 0.05;  ^*∗∗*^*P* < 0.01;  ^*∗∗∗*^*P* < 0.001.

Glutathione peroxidase 4 (GPX4), one of the members of the GPx family, was reported to be the most important inhibitory regulator of ferroptosis [22]. In this study, we found that HBx treatment decreased GPX4 expression and increased the acyl-CoA synthetase 4 (ACSL4) levels (an important ferroptosis-promoting factor) in both D-GalN-treated HL7702 cells and erastin-treated HL7702 cells. However, 500 nM Na_2_SeO_3_ treatment displayed the opposite effect compared with HBx treatment on the GPX4 and ACSL4 expression (Figures 6(a) and 6(b)). We then added the GPX4 inhibitor, RSL3, which was reported to enhance cell ferroptosis [22]. We found that 500 nM Na_2_SeO_3_ treatment almost totally reversed the increase in the HBx-related hepatotoxicity levels (Figures 6(c) and 6(d)). We then measured the correlation between the GPX4 and selenium levels in clinical serum samples. The data indicated that the serum GPX4 level in patients in the HBV^+^-HCC group was higher than that in the CHB group; also, both the HBV^+^-HCC and CHB groups had higher serum GPX4 levels compared with the HCs (Figure 6(e)). Furthermore, a significant negative correlation was observed between the GPX4 and selenium levels (Figure 6(f)). These results demonstrated that Na_2_SeO_3_–GPX4 axis inhibited the HBx-related hepatotoxicity in normal hepatocytes via ferroptosis.

### 3.6. Selenium Donor Inhibited the LPS/D-GalN-Induced Acute Liver Injury In Vivo

**Figure 7:**
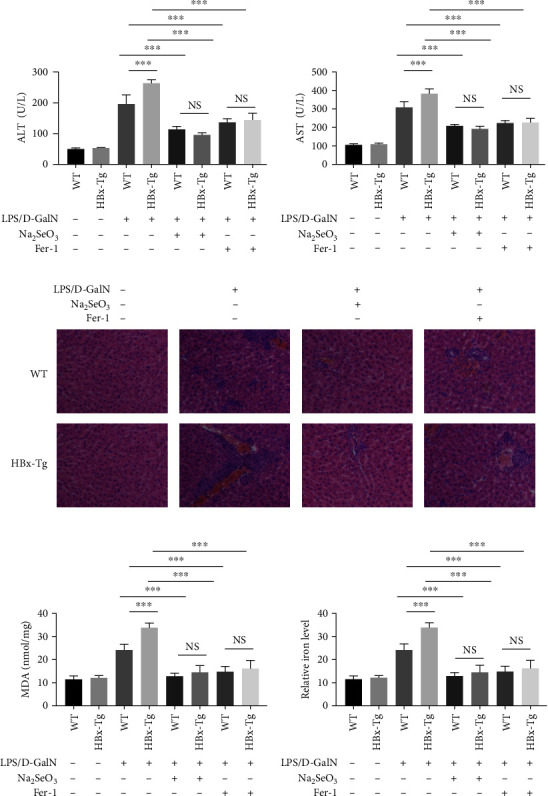
Treatment with Na_2_SeO_3_ or Fer-1 mitigated LPS/D-GalN-induced hepatic injury *in vivo*. The WT and HBx-Tg mice were subjected to control or LPS (0.25 mg/Kg) and D-GaIN (400 mg/Kg) treatment for 5 hr, as well as Na_2_SeO_3_ (3 mg/kg) or Fer-1 (10 mg/kg) administration. (a and b) Serum (a) ALT and (b) AST activities of each group. (c) Liver histopathology of each group. (d and e) Ferroptosis markers, including (d) MDA levels and (e) relative iron levels of each group. Each treatment group was composed of eight mice, which were kept in a cage.  ^*∗*^*P* < 0.05;  ^*∗∗*^*P* < 0.01;  ^*∗∗∗*^*P* < 0.001.

We next determined the effect of Na_2_SeO_3_ on the LPS/D-GalN-induced acute liver injury model in WT and HBx-Tg mice. The WT and HBx-Tg mice were subjected to control or LPS/D-GalN treatment, as well as Na_2_SeO_3_ or Fer-1 administration. The levels of plasma ALT and AST obviously increased 6 hr after the injection of LPS/D-GalN compared with those in the control group (Figures 7(a) and 7(b)). The ALT and AST levels were higher in HBx-Tg mice than in WT, but they decreased significantly to the levels observed after treatment with Na_2_SeO_3_ or Fer-1 (Figures 7(a) and 7(b)). The hematoxylin–eosin staining showed that the liver with LPS/D-GalN exposure presented marked histological abnormalities, including hepatocellular necrosis, disordered arrangement of hepatic lobules, and infiltration of inflammatory cells (Figure 7(c)). An increase in injury index, including inflammation, severe hepatocytes, swelling, and intrahepatic hemorrhage, was observed in HBx-Tg + LPS/D-GalN mice. These histological alterations induced by LPS/D-GalN were dramatically ameliorated in the two types of mice to the level observed after treatment with Na_2_SeO_3_ or Fer-1 (Figure 7(c)). Predictably, ferroptosis markers such as the MDA and iron levels in liver tissues were also potentiated in HBx-Tg + LPS/D-GalN mice, while Na_2_SeO_3_ or Fer-1 exhibited protective effects on these two markers (Figures 7(d) and 7(e)). These results confirmed that selenium donors could inhibit the LPS/D-GalN-induced acute liver injury *in vivo*.

## 4. Discussion

Although several previous studies indicated the antitumor properties of selenium in certain types of cancer, the novel data revealed that selenium could increase the risk of specific types of cancer. Therefore, whether a high level of selenium in patients helps improve the prognosis of antitumor treatment remains inconclusive [22–25]. Lubiński et al. [23] reported that a higher level of selenium was associated with a better prognosis of laryngeal cancer. They also demonstrated that higher levels of serum selenium might increase the survival rate in patients with breast cancer [24]. However, Kristal et al. [25] found that selenium intake did not improve prostate treatment outcomes. Furthermore, Vinceti et al. [26] demonstrated that selenium might even increase the risk of some cancer types, such as skin cancer. Vinceti et al. [26] pointed out that multiple limitations can lead to these differences, some of which may even be unavoidable. For example, Lubiński et al. [24] tested the selenium content in blood, while Kristal et al. [25] tested toenail selenium concentrations, both of which are limited by variations in the retention and the tissue-specific distribution of selenium not only according to its amount of exposure but also to its chemical forms and the concomitant exposure to other substances, such as methionine and heavy metals. Meanwhile, the differences in cancer species may be another important issue, as the content and function of selenium in the different organs varies significantly [26]. Therefore, exploring the impact of selenium on tumor occurrence and development should fully consider the influence of these conditions.

Several large-scale population studies showed a protective role of selenium in HBV and HCC. Yu et al. [27] conducted a selenium intervention test in five townships of Qidong, China (this area has the second-highest rate of HCC in China). Table salt fortified with 15 ppm sodium selenite was provided to the general population of 20,847 persons in one town. Those in the other four townships with similar primary liver cancer rates served as controls and received plain table salts. The 8-year follow-up study showed that the HCC prevalence rate was reduced by 35.1% in the selenium-supplemented population compared with the control population. Also, they found that HBV carriers in the selenium-supplemented group had a lower risk of developing HCC [27]. Similar results were also obtained in some other clinical trials, and Darvesh and Bishayee [28] summarized these results in their study. These studies may also inevitably have limitations, such as testing sample singularity (only blood was tested) and testing population singularity (only Han race was tested), but their results provide a good reference for our exploration. In the present study, we tested the serum selenium levels in three groups, revealing that the selenium levels were lower in patients in the HBV^+^-HCC group than in the CHB group. Also, patients in both HBV^+^-HCC and CHB groups had lower selenium levels compared with HCs. Furthermore, patients in the HBV^+^-HCC group with higher serum selenium levels had a better tumor size and lymphatic metastasis with lower overall and disease-free survival rates. Our results are consistent with previous studies [27], which increases the credibility that selenium can indeed affect the pathogenesis of HBV-related HCC.

We next experimentally confirmed part of the clinical results and preliminarily explored the molecular mechanisms involved. The cell-based data indicated that selenium limited HBV replication, which was consistent with the previous findings [18]. Furthermore, we found that selenium donor Na_2_SeO_3_ inhibited HBV replication by decreasing CP-Luc and XP-Luc activities, and this mode of action was similar to the effect of interleukin (IL)-18, as described by Zhang et al. [29]. Zhang et al. [29] demonstrated that inhibiting the HBV core gene and X gene promoters by IL-18 depended mainly on inflammatory cytokines and the NF-*κ*B pathway. Our study demonstrated that selenium donors inhibited HBV replication via apoptotic cell death. Selenium has been reported to regulate cellular apoptosis through multiple mechanisms, and the regulatory roles are closely related to its form and concentration. In this study, we found that 5 *μ*M Na_2_SeO_3_ could target cIAPs, which might be the main mechanism of its role in apoptosis regulation.

Both the inflammatory and cell death pathways are “double-edged swords” [29]. Although programmed cell death–like apoptosis plays an inhibitory role in HBV replication and related HCC development, excessive cell death in normal hepatocytes could cause hepatotoxicity, which would also lead to tumor formation. The clinical data of this study showed that high level of selenium was associated with better HCC prognosis and low level of hepatotoxicity, which could not be achieved via any other single regulation of the apoptosis pathway. This was because selenium might regulate different cell death mechanisms in different stages of HCC development. We found that, in the hepatotoxicity cell model, 500 nM of Na_2_SeO_3_ protected the cells independent of apoptosis, but by inhibiting ferroptosis.

Ferroptosis is an iron-dependent form of regulated necrosis [10, 11]. It is implicated in various human diseases, including organ damage and cancer [22]. As a recently discovered new cell death mode, ferroptosis has been found to mediate HBx-induced hepatotoxicity [8]. The inhibition of ferroptosis using a selenium donor has not been clearly reported, but it can be inferred from some reported mechanisms. For example, the selenium donor could decrease the production of reactive oxygen species, promote autophagy, and induce GPx activity [13, 16], which were the upstream regulatory factors of ferroptosis [10, 11]. Besides GPX4 activity, our findings indicated that selenium could also regulate GPX4 expression. The GPX4 activity required selenocysteine; previous studies revealed that selenium regulated the GPX4 activity. However, a recent study demonstrated that selenoproteins were dispensable for cell viability and ferroptosis-provided partial GPX4 activity was retained [30]. These results suggested that regulating GPX4 expression using selenium might play a significant role in cell ferroptosis.

An anti-HCC therapy targeting ferroptosis has broad application prospects. Sorafenib is an approved drug to treat HCC and was known to induce ferroptosis of cells [31]. Although sorafenib has multiple targets, some evidence suggests that sorafenib resistance in HCC is closely related to ferroptosis [32]. Therefore, theoretically, the intake of selenium supplements may improve the efficacy of sorafenib and alleviate sorafenib resistance by targeting ferroptosis.

## 5. Conclusions

This study provides several important insights for subsequent clinical research. First, we confirmed that an inorganic selenium donor, Na_2_SeO_3_, has the potential to treat HBV-associated HCC. Second, we demonstrated that the dosage of Na_2_SeO_3_ was closely related to its function, which could be referenced in clinical trials. Third, we identified potential targets for Na_2_SeO_3_, which helps to monitor the efficacy of Na_2_SeO_3_ in clinical practice by testing the levels of these indicators. However, there are also several limitations in this study, such as small sample size, testing sample singularity (only blood was tested), and testing population singularity (only Han race was tested). In addition, several laboratory's conclusions still need to be validated with clinical data. Therefore, further studies need to be conducted in wider cohorts to verify the aforementioned findings.

## Figures and Tables

**Table 1:** Demographics and clinical features of the HBV^+^-HCC patients, CHB patients and the healthy controls.

Clinical characteristics	HC group (*n* = 45)	CHB group (*n* = 45)	*P*_1_^a^ value	HBV^+^-HCC group (*n* = 45)	*P*_2_^b^ value	*P*_3_^c^ value
Gender						
Male	30	33	0.490	36	0.153	0.455
Female	15	12		9		
Age						
>55	21	23	0.673	20	0.832	0.527
≤55	24	22		25		
HBV-DNA positive						
Yes	–	36		34		0.612
>10^3^ copies/mL						
No	–	9		11		
Anti-HBV treatment						
Yes	–	24		26		0.6714
No	–	21		19		
Tumor size						
>5	–	–	–	20	–	0.670
≤5	–	–		25		
Stage						
I/II	–	–	–	23	–	0.834
III/IV	–	–		22		
Lymphatic metastasis						
Yes	–	–	–	19	–	0.516
No	–	–		26		

^a^ *P*_1_ was calculated by comparing the difference between CHB patients with the HCs group; ^b^*P*_2_ was calculated by comparing the difference between HBV^+^-HCC patients with the HCs group; ^c^*P*_3_ was calculated by comparing the difference between HBV^+^-HCC patients with CHB group.

**Table 2:** Serum selenium levels in HBV^+^-HCCs group and CHB group.

Clinical characteristics	CHB group (*n* = 45)			HBV^+^-HCC group (*n* = 45)		
	Se low (*n* = 22)	Se high (*n* = 23)	*P* value	Se low (*n* = 22)	Se high (*n* = 23)	*P* value
Age						
>55	8	14	0.100	12	8	0.182
≤55	14	9		10	15	
Gender						
Male	14	19	0.150	3	6	0.297
Female	8	4		19	17	
HBV-DNA positive						
Yes	19	17	0.297	17	17	0.793
>10^3^ copies/mL						
No	3	6		5	6	
Anti-HBV treatment						
Yes	16	8	0.0108	16	10	0.0471
No	6	15		6	13	
Tumor size						
>5	–	–	–	14	6	0.0113
≤5	–	–		8	17	
Stage						
I/II	–	–	–	8	15	0.0529
III/IV	–	–		14	8	
Lymphatic metastasis						
Yes	–	–	–	14	5	0.0045
No	–	–		8	18	

## Data Availability

The data that support the findings of this study are available from the corresponding author upon reasonable request.

## Abbreviations

HCC: Hepatocellular carcinoma
HBV: Hepatitis B virus
HCs: Healthy cases
CHBs: Chronic hepatitis B virus infection patients
HBx: HBV X protein
HBsAg: HBV surface antigen
HBeAg: HBV e antigen
SPI: Surface promoter I
SPII: Surface promoter II
CP: Core promoter
ROC: Receiver operating characteristic curve
AUC: Area under the curve
cIAPs: Cellular inhibitor of apoptosis proteins
GPXs: Glutathione peroxidases
MDA: Malondialdehyde
ACSL4: cyl-CoA synthetase 4
GPX4: Glutathione peroxidase 4.
